# The Impact of Infections on the Progression of Chronic Kidney Disease

**DOI:** 10.3390/medicina59101836

**Published:** 2023-10-15

**Authors:** Ioana Dicu-Andreescu, Cristina Căpușă, Liliana Gârneață, Otilia-Andreea Ciurea, Irinel-Gabriel Dicu-Andreescu, Elena-Alexandra Ungureanu, Denis-Valentin Vlad, Antonia-Constantina Vișan, Victor-Gabriel Ungureanu, Violeta-Valentina Vlad, Patrick-Christian Vasioiu, Elis-Mihaela Ciutacu, Mihaela Neicu, Mircea Penescu, Constantin Verzan

**Affiliations:** 1 Faculty of Medicine, “Carol Davila” University of Medicine and Pharmacy, Street Eroii Sanitari No. 8, Sector 5, 050474 Bucharest, Romaniaandreescugabriel43@gmail.com (I.-G.D.-A.); visanantoniaa@gmail.com (A.-C.V.);; 2 Nephrology Department, “Dr. Carol Davila” Clinical Hospital of Nephrology, Street Calea Griviței No. 4, Sector 1, 010731 Bucharest, Romania

**Keywords:** infections, chronic kidney disease, progression of chronic kidney disease, independent predictors of the evolution of chronic kidney disease

## Abstract

*Background and Objective*: Infectious diseases continue to be a global burden and their impact is even worse if the patients already have other comorbidities. Because chronic kidney disease is very frequent, affecting 10% of the population, our study aims to explore the impact that infectious events have on its progression. *Material and Methods*: This is a retrospective, observational study based on a cohort of 238 dialyzed patients from the Nephrology Clinic of “Dr. Carol Davila” Clinical Hospital of Nephrology, Bucharest, who were followed from their first visit for five years, between 1 January 2007 and 1 January 2022. For each of them, the presence of an infectious event and the moment of the initiation of dialysis were recorded. *Results*: Statistical analysis showed that the patients who had at least one infectious episode were older (*p* = 0.004), their hemoglobin and lymphocytes were significantly lower (*p* = 0.03 and *p* = 0.02, respectively) and the time until the initiation of dialysis was lower (*p* = 0.007). Also, the preservation of kidney function was influenced by the number and the severity of infectious episodes. In the univariate Cox model, the following variables were associated with increased risk of dialysis: advanced age (*p*: 0.009; HR: 1.021; CI: 1.005 to 1.036), low hemoglobin (*p*: 0.001; HR: 0.861; CI: 0.786 to 0.943), previous diagnosis of chronic obstructive pulmonary disease (*p*: 0.002; HR: 2.467; CI: 1.376 to 4.424), presence of hematuria (*p*: 0.03; HR: 1.604; CI: 1.047 to 2.457) and increased values of proteinuria (*p*: 0.01; HR: 1.122; CI: 1.028 to 1.224) and of serum creatinine measured both at the time of the first visit and at the time of each infectious event (*p*: <0.001; HR: 1.262; CI: 1.141 to 1.396). Also, the presence of an infectious episode was associated with a 1.7-fold increase in the risk of dialysis initiation. The independent predictors of survival identified by the multivariate Cox model were age (*p*: 0.004; HR: 1.034; CI: 1.010–1.058), serum creatinine (*p*: <0.001; HR: 1.421; CI: 1.203 to 1.658) and proteinuria (*p*: <0.001; HR: 1.241; CI: 1.126 to 1.369) at the time of enrollment, but also the presence of an infectious episode during the patient’s evolution (*p*: 0.04; HR: 1.705; CI: 1.013 to 2.868). *Conclusions*: In the evolution of patients with chronic kidney disease, an active search for individual factors favoring the occurrence of infectious episodes should be taken into consideration to prevent a faster progression toward end-stage kidney disease.

## 1. Introduction

Chronic kidney disease (CKD) affects more than 10% of the population and is defined by the Kidney Disease Improving Global Outcomes (KDIGO) guidelines as the presence of structural abnormalities, most often represented by albuminuria, or functional abnormalities, with a glomerular filtration rate (GFR) below 60 mL/min/1.73 m^2^, which are present for at least 3 months [1,2]. The most important risk factors for CKD are hypertension, obesity, diabetes and smoking [3].

However, another potential risk factor, often overlooked, is represented by infections, especially in the context of studies showing that patients with CKD have a particularly high risk of hospitalization and, consequently, of infection. One such study was conducted by Ishigami in 2019 and claimed that the risk of hospitalization due to infectious causes increases by 50% in stage G3 of CKD and by two to three times in stages G4 and G5 compared to stage G2 [4]. Moreover, the risk of death from an infectious event increases three times in the G3b stage compared to the G2 stage, with the cause being represented by the immunological, inflammatory and metabolic changes associated with the progression of renal function deterioration, which place the infectious diseases on the list of causative agents of death in patients with CKD [5,6]. In addition, the most important and well-known predictor factor for the progression of CKD is proteinuria, and a fact that should be noted is that between proteinuria and the risk of infectious events, a directly proportional relationship was found, especially in older patients [7,8,9].

Because of these aspects, it becomes necessary to evaluate if only CKD favors the infections, or if the infectious events are also independent risk factors for the progression of the deterioration of kidney function.

## 2. Materials and Methods

This is a retrospective, observational study in which we analyzed a cohort of 238 patients from the Nephrology Clinic of “Dr. Carol Davila” Clinical Hospital of Nephrology, Bucharest. We looked for patients who were on dialysis on 1 January 2022 and we followed them from the moment of their first visit to the clinic to the moment of the initiation of the kidney replacement therapy (KRT). However, because each of them had a different time until they reached the end point, defined as the initiation of the KRT, and thus the follow-up period would not have been homogenous, we chose to focus on their first five years of periodic controls to assess who needed KRT and, especially, what infectious events occurred during this time frame.

We chose this study design for a few reasons: the most important is that we had easy access to all the information needed for our study, with our aim being just to test what occurs to a patient with an infectious event in the past regarding the progression toward KRT, a fact that does not necessarily need special parameters or advanced laboratory tests that could have been missed in the last 15 years. In addition, in this way, we were able to create a large enough cohort in a relatively short time, which allowed us to carefully analyze the follow-up parameters, including what we were particularly interested in, namely the occurrence of infectious episodes.

Initially, we included all the patients that were dialyzed on 1 January 2022, and the cohort comprised 404 persons. However, after careful analysis, we excluded 150 of them either because they did not come periodically for the control, or the time intervals between the check-ups were over six months, which was considered to be too much to obtain relevant information about an infectious event. In addition, those under the age of 18 at the time of first presentation to the clinic and those with a kidney transplant, which meant another 16 patients, were also excluded, and, therefore, the final cohort was represented by 238 patients. The first visit of the patients in the clinic ranged from 1 April 2007 to 31 December 2016, which allowed for each of them to have five years of follow-up until 1 January 2022, considered the end of this study. Also, the end point was considered either the initiation of KRT, if this occurred before completing five years of follow-up, or the end of this time frame, so that the follow-up period was homogenous.

The following parameters were recorded: age, sex and comorbidities, also evaluated using the Charlson score; complete blood count and values of the inflammation parameters at the first visit to the hospital; the presence of infectious episodes, classified according to severity into mild, moderate and severe; the microorganisms involved; the prescribed antibiotic treatment; episodes of septic acute kidney injury; and the need to initiate kidney replacement therapy (KRT). The information about the infectious events was obtained in multiple ways: the most frequent one is that the patients presented directly in our clinic for investigations and treatment when such an event occurred and we used our own clinical and laboratory results; another one is by using the information provided to the patients when he/she was discharged from other hospitals, information that has been recorded in our documents at each visit.

The main hypothesis of this study is that an infectious episode occurring in a patient with CKD leads to a faster progression toward end-stage kidney disease and the need for KRT initiation, with all the consequences involved. To test this hypothesis, we evaluated the statistically significant differences between those with and without infections using the Mann–Whitney U test and Fisher’s exact test, we built Kaplan–Meier survival curves, we performed univariate Cox regression to identify the risk factors for KRT initiation, and last, but not least, we included the significant variables obtained from the univariate analysis in a multivariate Cox model to identify the independent predictors of KRT initiation in the cohort.

## 3. Results

With the exception of hemoglobin, reported as the mean and standard deviation (SD), all the other variables had an abnormal distribution and are reported as the median and inter-quartile range (IQR). The first 11 variables presented in the table below have the values recorded at the time of the first visit. Also, in the last column, the *p*-value measures the differences between groups and was obtained using the statistical tests detailed below. The end point is defined as the moment at which KRT is initiated during the follow-up period (Table 1).

The first step was to analyze the differences between those with and without infections using the Mann–Whitney U test, to compare the values of continuous variables, and Fisher’s exact test, to compare the values of categorical data. We used the Mann–Whitney U test because the data in our cohort had an abnormal distribution.

The following variables were significantly different in the group of the patients that experienced at least one infectious event: age was significantly higher: *p* = 0.004; hemoglobin and lymphocytes were significantly lower: *p* = 0.03 and *p* = 0.02, respectively; females were more likely to have an infectious event: *p* = 0.01; hematuria and leukocyturia at the time of the first visit were also more frequent: *p* = 0.01 and *p* = <0.001, respectively; the time until the end point was lower: *p* = 0.007; the initiation of KRT was more frequent: *p* = 0.004; and, as expected, the final glomerular filtration rate was lower in the group with infections: *p* = 0.017. A crucial aspect that should be emphasized is that there were no differences between groups regarding the initial stage of CKD, the etiology of CKD and the risk group calculated based on serum creatinine and proteinuria, as a heterogeneous distribution of these variables could have led to erroneous conclusions, because it is expected for a person in a more advanced stage, in a higher risk group or with a specific etiology of CKD to reach the end point faster. Also, except for COPD, which was more frequent in those who underwent an infectious episode, *p* = 0.008, the other comorbidities evaluated (hypertension, stroke, diabetes mellitus, malignancy, heart failure and chronic venous insufficiency) were equally distributed in the groups too.

In our cohort, 117 patients had at least one infectious episode. To be able to assess the impact on kidney function as precisely as possible, the infections were classified according to severity, using the following criteria (Table 2):

1. Mild infections: did not require hospitalization for treatment, were self-limited or required a short course of antibiotic therapy (less than 5 days). The inflammatory syndrome was minimal or absent. Among these, the following were identified in our patients: asymptomatic bacteriuria, cystitis, dental infections, uncomplicated upper-respiratory-tract infections and gastritis.
2. Moderate infections: inflammatory syndrome present (CRP between 10 and 30 UI/mL, leukocytosis), required hospitalization or occurred during hospitalization and had the potential for progression in the absence of antibiotic treatment. Among them, the following were identified in our group: orchiepididymitis, prostatitis and post-operative infections of soft tissue.
3. Severe infections: had significant inflammatory syndrome (CRP over 30 UI/mL, leukocytosis/leukopenia), required hospitalization and prolonged antibiotic therapy, and patients often presented with altered general condition. Severe infections are also considered life-threatening. Among them, the following were identified in our patients: sepsis, endocarditis, cholecystitis, enterocolitis due to *Clostridioides difficile*, pyelonephritis and acute lower-respiratory-tract infections. In our study, we excluded viral infections such as those caused by hepatitis B or C or HIV due to the multitude of different possible patterns of glomerular injury associated with them, each with a different evolution toward end-stage kidney disease. On the other hand, viral infections causing upper- or lower-respiratory-tract infections were not excluded, but we could not properly identify which virus was involved specifically.

In the evolution of each patient, the occurrence of a maximum of three infectious episodes was followed, documented by the type of infection, the microorganism involved, when it was possible, and the impact on kidney function estimated based on serum creatinine. For those patients in whom more than three infectious episodes were identified (24 patients), a separate column was filled in, without further details.

Table 3 shows in descending order of frequency the types of infections identified in the cohort, as well as the most common microorganisms involved, separated by episode.

**Figure 1:**
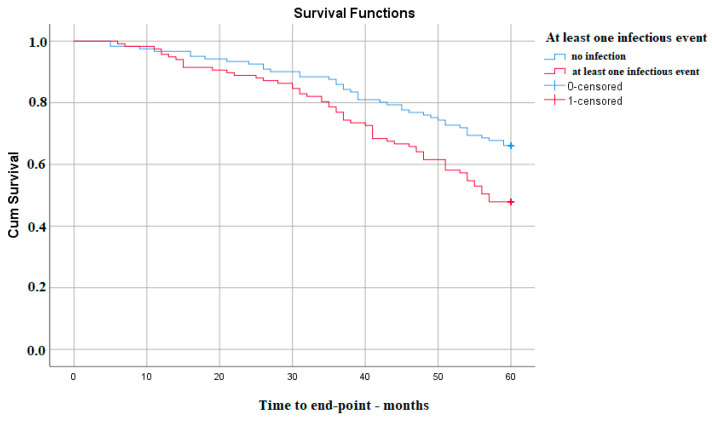
Kaplan–Meier survival analysis for at least one infectious event in the follow-up period.

Kaplan–Meier survival analysis reported that there is a significant difference in 5-year survival without the need for KRT between those with and without infections, p(Log-Rank)—0.005, much lower in the group that experienced at least one infectious episode (Figure 1).

**Figure 2:**
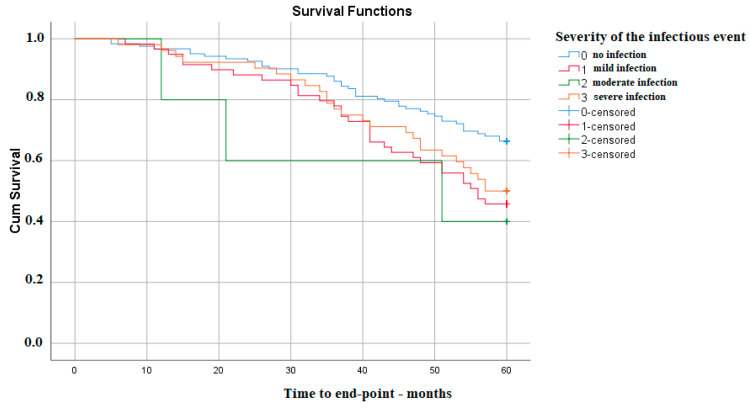
Kaplan–Meier survival analysis—severity of infection.

Also, the severity of infectious events is associated with a lower survival time of kidney function. The differences appear between the absence of any type of infection and the occurrence of a mild (p Log Rank 0.007) or severe one (p Log Rank 0.043). Interestingly, in this cohort, there was no significant difference regarding moderate infectious events, most probably because the number of these was not large enough (Figure 2).

**Figure 3:**
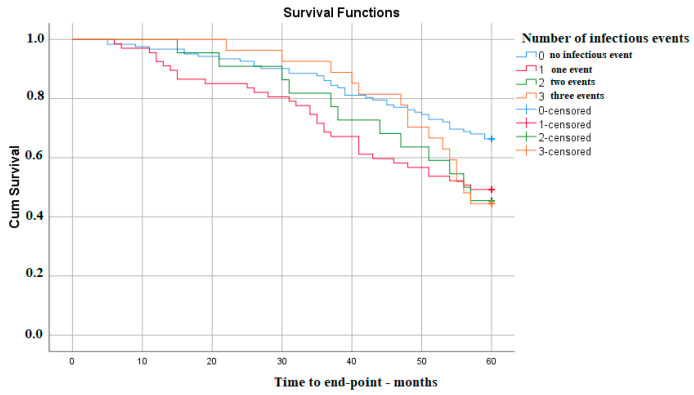
Kaplan–Meier survival analysis—number of infections.

On the other hand, the survival of kidney function does not appear to decrease with the number of infectious events, with the only significant difference being between no infection during follow-up and only one such event—p Log Rank 0.010 (Figure 3).

Last, but not least, in our cohort, the presence of a septic acute kidney injury was not associated with a lower kidney function survival during follow-up, most probably due to the small number of such events in our group—p Log Rank 0.072.

To determine the risk of each variable being associated with reaching the end point faster, the univariate Cox model was used, with the following results: from the values recorded at the moment of the first visit in the clinic, advanced age, low hemoglobin level, the diagnosis of chronic obstructive pulmonary disease, the presence of hematuria, as well as increased values of proteinuria/day and serum creatinine were associated with increased risk of KRT initiation during the 5-year follow-up period. Also, the presence of an infectious episode during follow-up was associated with a 1.7-fold increase in the risk of initiating KRT. Among the types of infections registered in the cohort, only the first episode showed significant associations, as follows: lower-urinary-tract infections increase the risk by 1.9 times and acute lower-respiratory-tract infections increase the risk by 3.6 times. Also, increased values of serum creatinine at the time of the onset of infections are associated with an increased risk of KRT (Table 4).

Lastly, these variables were included in a multivariate Cox model to be able to determine the independent predictors of the survival of kidney function. After analysis, it was found that they are represented by serum creatinine and proteinuria measured at the time of enrollment, which are also validated, well-known predictors of CKD progression, but also the age and the presence of an infectious episode during the patient’s evolution, without, however, the ability to specify, based on the data of this cohort, which type of infection predisposes the patient to reaching end-stage kidney disease quicker. The precise statistical values are presented in Table 5, comparatively between those from the univariate Cox model and those from the multivariate Cox model.

## 4. Discussion

This study has confirmed that an infectious event that appears in the evolution of a patient with chronic kidney disease has the independent capacity to induce a more rapid progression toward end-stage kidney disease and, consequently, the necessity of KRT. Another important aspect of this study is that there are no differences between the stages of the CKD, the risk group or the etiology of CKD at the time of the first visit between those who experienced an infectious event and those who did not, a fact that allows us to draw correct conclusions.

On the other hand, this article has a few limitations.

First of all, the cohort is not very large, which does not allow a better exploration of the impact on the survival of the kidney function of each type of infection and, therefore, of the microorganisms involved. However, it allowed a good follow-up of the patients, as well as of the treatments performed and biological parameters. Also, because this study is unicentric, carried out in a tertiary center focused on the study of kidney diseases and includes a reasonable number of patients, it provides results that can be extrapolated for the kidney and infectious pathology in this area of the country.

Secondly, being a retrospective study, we can assume that the patients experienced more infectious events that could not have been captured, in both of the groups, which, again, can have a significant impact. In addition, the treatment, even though we recorded it for each infectious event, could not be statistically evaluated, because the compliance of the patients could not be controlled at an acceptable level of trust. Because of this aspect, in many cases, it is possible that the progression of CKD to be due to the persistent inflammatory state induced by chronic infection.

Last, but not least, the nephrotoxicity of antibiotics can play a key role in patients with infectious events, because studies argue that they are prescribed in up to 64% of cases in much higher doses and they can affect the kidney through multiple routes: interstitial nephritis, direct tubular toxicity, immune dysfunction and decreased renal perfusion [9,10].

The mechanisms that lie behind the fact that an infectious event can lead to a faster progression toward ESKD are yet to be fully understood, but it is supposed that the main causes are related to persistent infection, genetic alterations of the complement pathways, tubulointerstitial changes and pre-existent kidney damage [11]. Also, a series of biomarkers have been proposed to help in recognizing which patients should be monitored more closely because of an increased risk for progression, like nephritis-associated plasmin receptor and interstitial alfa-smooth muscle actin [11]. The first was initially isolated from group A streptococci and it is involved in maintaining glomerular inflammation through local activation of the complement system, and the second is a marker of tubulointerstitial fibrosis [11,12]. In addition, regarding urinary tract infections, a key element in pathophysiology that predisposes the patients to this type of infection is represented by genetic polymorphisms of Toll-like receptors (TLRs) [13,14]. Of these, the most important are TLR2, TLR4 and TLR5 [15]. Moreover, genetic alterations of vascular endothelial growth factor A (VEGFA) and transforming growth factor beta 1 (TGFβ1) are related to kidney scarring and progressive kidney disease after urinary tract infections, especially those associated with vesicoureteral reflux [16].

Also, this study confirmed the importance of the classical predictor factors for the progression of chronic kidney disease—serum creatinine and proteinuria at the baseline. Of these two predictors, proteinuria is linked to the activation of inflammatory and fibrotic pathways that lead to glomerulosclerosis and advanced CKD, but also to an increased risk of infection [17]. In addition, a systematic review analyzed the importance of pre-operative proteinuria in patients who underwent partial or radical nephrectomy for kidney cancer, and it concluded that it can increase the long-term kidney impairment and the risk for acute kidney injury after surgical intervention [18]. Moreover, proteinuria seemed to have a prognostic capacity independently of that of estimated GFR, making it a valuable tool in the assessment of the overall mortality risk, especially during pre-operative states [18].

Other useful markers that evaluate the progression of CKD have been proposed, like cystatin C; neutrophil-gelatinase-associated lipocalin (NGAL); kidney injury molecule 1 (KIM-1), also considered one of the links between acute kidney injury and CKD; or liver-type fatty acid binding protein (L-FABP), which is correlated with the tubulointerstitial damage, but also claimed to be even more sensitive than proteinuria in predicting the progression of CKD, although with a lower specificity [19,20,21,22]. Further studies are needed to confirm these biomarkers and also to search for others, in an attempt to find the factor with the greatest capacity of prediction, or to build a model composed of few such biomarkers that reinforce each other in order to better understand and predict the evolution toward ESKD.

## 5. Conclusions

The results of this study demonstrate that the presence of an infectious episode in the evolution of a patient with CKD is an independent predictor of the survival of kidney function, along with the well-known, classical predictor factors—serum creatinine and proteinuria at the time of enrolment. Also, the severity and the number of infectious events influence the preservation of kidney function, with mild and severe infections having the greatest impact. On the other hand, the occurrence of septic acute kidney injury does not seem to have an impact on the evolution of kidney function degradation, a fact that can be explained by the small number of these events in our cohort.

Correlating these aspects with the one presented in the literature, according to which the risk of death from an infectious cause increases as kidney function deteriorates, all patients have to be instructed not to treat infectious episodes superficially, and, from the moment they occur, be followed more closely and at shorter intervals. Patients should also be advised to stop any nephrotoxic medication/supplement to minimize the negative impact of cumulative offending factors, and clinicians should be advised to carefully adjust the doses of antibiotics to the kidney function. In addition, supplementary tests should be taken into consideration after the initial infectious episode to ensure that the infection was really cured.

Further research is needed to confirm the findings of our study and, in the future, to provide better insight into the mechanisms that lead to the progression of CKD and into the most useful biomarkers that can be used to evaluate the patients.

## Figures and Tables

**Table 1:** General characteristics of the patients.

	Total (238)	With Infections (117)	No Infections (121)	*p*-Value
**Age, years, median (IQR)**	60 (18)	62 (20)	57 (18)	0.004
**Sex M, n (%)**	130 (54.6)	54 (46.1)	76 (62.8)	0.01
**Hemoglobin, g/dL, mean (SD)**	11.7 (2.20)	11.3 (2.18)	12.05 (2.18)	0.03
**Leukocytes/mL, median (IQR)**	7700 (3025)	7700 (3500)	8000 (2800)	0.47
**Neutrophils/mL, median (IQR)**	5100 (2525)	4900 (2850)	5400 (2050)	0.19
**Lymphocytes/mL, median (IQR)**	1850 (800)	1800 (800)	2000 (800)	0.02
**CRP, UI/mL, median (IQR)**	5 (9)	5 (10)	5 (9)	0.31
**Fibrinogen, mg/dL, median (IQR)**	573 (224)	586 (234)	560 (201)	0.06
**Serum albumin, g/dL, median (IQR)**	4.27 (0.59)	4.28 (0.62)	4.22 (0.59)	0.87
**Hematuria, n (%)**	149 (62.6)	82 (70)	67 (55.3)	0.01
**Leukocyturia, n (%)**	126 (52.9)	83 (70.9)	43 (35.5)	<0.001
**SCr_M0, mg/dL, median (IQR)**	2.3 (1.55)	2.17 (1.77)	2.39 (1.32)	0.708
**eGFR_M0, mL/min, median (IQR)**	27 (21)	27 (24.2)	29 (18.8)	0.431
**SCr_inf1, mg/dL, median (IQR)**		2.83 (3.22)		
**eGFR_inf1, mL/min, median (IQR)**		19.1 (19.93)		
**SCr_inf2, mg/dL, median (IQR)**		3.35 (3.21)		
**eGFR_inf2, mL/min, median (IQR)**		15 (19.5)		
**SCr_inf3, mg/dL, median (IQR)**		3.43 (2.9)		
**eGFR_inf3, mL/min, median (IQR)**		14 (15)		
**Final eGFR, mL/min, median (IQR)**	9 (14)	69 (8.5)	13 (18)	0.017
**Time M0_endpoint, months, median (IQR)**	60 (19)	57 (23)	60 (11)	0.007
**Time M0_inf1, months, median (IQR)**		4 (23)		
**Time inf1_inf2, months, median (IQR)**		17 (23)		
**Time inf2_inf3, months, median (IQR)**		6 (14)		
**Time inf3_endpoint, months, median (IQR)**		15 (20)		
**Severity of infection, n (%)**		1. Mild—60 (51.2) 2. Moderate—5 (4.2) 3. Severe—52 (44.4)		
**At least two severe infections, n (%)**		11 (9.4)		
**More than three infections, n (%)**		24 (20.5)		
**Septic AKI, n (%)**		0 episodes—103 (88) 1 episode—14 (11.9)		
**KRT during the follow-up period, n (%)**	102 (42.8)	61 (52.1)	41 (33.8)	0.004
**Etiology of CKD (%)**	1. Benign nephrosclerosis (28.1) 2. Primary glomerulopathies (22.6) 3. Diabetic nephropathy (13.4)	1. Benign nephrosclerosis (26.4) 2. Primary glomerulopathies (17.9) 3. Diabetic nephropathy (10.2)	1. Benign nephrosclerosis (29.7) 2. Primary glomerulopathies (27.2) 3. Diabetic nephropathy (16.5)	0.10
**Stage of CKD at the first visit, n (%)**	G1: 3 (1.2)	G1: 0 (0)	G1: 3 (2.4)	0.46
	G2: 16 (6.7)	G2: 9 (7.7)	G2: 7 (5.8)	
	G3a: 23 (9.6)	G3a: 14 (11.9)	G3a: 9 (7.4)	
	G3b: 62 (26)	G3b: 25 (21.3)	G3b: 37 (30.5)	
	G4: 96 (40.3)	G4: 49 (41.8)	G4: 47 (38.8)	
	G5: 38 (15.9)	G5: 20 (17)	G5: 18 (14.8)	
**Risk group, n (%)**	1. Low—5 (2.1) 2. Moderate—5 (2.1) 3. High—25 (10.5) 4. Very high—203 (85.2)	1. Low—2 (0.8) 2. Moderate—3 (1.2) 3. High—13 (5.4) 4. Very high—99 (84.6)	1. Low—3 (2.4) 2. Moderate—2 (1.6) 3. High—12 (9.9) 4. Very high—104 (85.9)	0.92
**Hypertension, n (%)**	220 (92.4)	110 (94)	110 (90.9)	0.36
**Diabetes mellitus, n (%)**	89 (37.3)	41 (35)	48 (39.6)	0.46
**Stroke, n (%)**	19 (7.9)	10 (8.5)	9 (7.4)	0.75
**Malignancies, n (%)**	46 (19.32)	20 (17.09)	26 (21.4)	0.39
**COPD, n (%)**	16 (6.8)	13 (11.1)	3 (2.4)	0.008
**Heart failure, n (%)**	73 (30.6)	33 (28.2)	40 (33)	0.41
**Chronic venous insufficiency, n (%)**	22 (9.2)	8 (6.8)	14 (11.5)	0.20
**Charlson score, median (IQR)**	6 (4)	6 (4)	6 (4)	0.46

n—number of cases; M—male; SD—standard deviation; IQR—interquartile range; CRP—C reactive protein; SCr—serum creatinine; eGFR—estimated glomerular filtration rate; M0—baseline level; inf1—first infection; inf2—second infection; inf3—third infection; AKI—acute kidney injury; CKD—chronic kidney disease; COPD—chronic obstructive pulmonary disease.

**Table 2:** Assessment of the severity of infections and their classification.

Total: 117	Mild Infections-60	Moderate Infections-5	Severe Infections-52
Inflammatory syndrome	Minimum/Absent	Moderate	Severe
Antibiotics	Short course (5 days)	Sometimes a prolonged course	Often prolonged course
Hospitalization	No need	Can be required Infections that occurred during hospitalization	Required
Evolution/Risks	Self-limited	Potential for progression	Potentially life-threatening in the absence of treatment
	-asymptomatic bacteriuria	-orchiepididymitis	-sepsis
	-cystitis	-prostatitis	-endocarditis
	-dental infections	-post-operatory infections of soft tissue	-cholecystitis
	-upper-respiratory-tract infections		-enterocolitis
	-gastritis		-pyelonephritis
			-lower-respiratory-tract infections

**Table 3:** Characteristics of the infectious episodes.

First Infectious Episode (117 Cases)	N (%)	The Most Common Microorganism Identified (%)
Lower UTIs	67 (57.2)	*E. coli* (43.2)
Upper UTIs	20 (17)	*Klebsiella* spp. (20)
Acute lower-respiratory-tract infections	15 (12.8)	*Candida* spp. (26.6)
Enterocolitis	4 (3.4)	*Cl. difficile* (75)
Sepsis	2 (1.7)	*Klebsiella* spp. (100)
Soft tissue	2 (1.7)	*Staphylococcus* spp. (50)
Acute upper-respiratory-tract infection	1 (0.8)	
Gastritis	1 (0.8)	*H. pylori* (100)
Endocarditis	1 (0.8)	
Dental infection	1 (0.8)	
Orchiepididymitis	1 (0.8)	
Cholecystitis	1 (0.8)	
**Second infectious episode** **(49 cases)**	**N (%)**	**The most common microorganism identified (%)**
Lower UTIs	28 (57)	*E. coli* (35)
Upper UTIs	12 (24)	*E. coli* (58)
Acute lower-respiratory-tract infections	4 (8)	*Klebsiella* spp. (50)
Enterocolitis	2 (4)	*Cl. difficile* (50)
Acute upper-respiratory-tract infection	1 (2)	
Soft tissue	1 (2)	
Dental infection	1 (2)	
**Third infectious episode** **(27 cases)**	**N (%)**	**The most common microorganism identified (%)**
Lower UTIs	21 (7)	*E. coli* (33)
Enterocolitis	2 (7)	*Cl. difficile* (100)
Upper UTIs	1 (3)	
Acute lower-respiratory-tract infections	1 (3)	*Klebsiella* spp. (100)
Soft tissue	1 (3)	
Acute upper-respiratory-tract infections	1 (3)	

N—number of cases; UTIs—urinary tract infections; *E. coli*—*Escherichia coli*; *Cl. difficile*—*Clostridioides difficile*; *H. pylori*—*Helicobacter pylori*; spp—species.

**Table 4:** Results of the univariate Cox model.

Variable	HR	CI	*p*-Value
**Age (M0)**	1.021	1.005–1.036	0.009
**Hemoglobin (M0)**	0.861	0.786–0.943	0.001
**Hematuria (M0)**	1.604	1.047–2.457	0.03
**Proteinuria (M0)**	1.122	1.028–1.224	0.01
**Serum creatinine (M0)**	1.262	1.141–1.396	<0.001
**Serum creatinine at inf1**	1.150	1.081–1.224	<0.001
**Serum creatinine at inf2**	1.413	1.188–1.679	<0.001
**Serum creatinine at inf3**	1.506	1.126–2.015	0.006
**Presence of an infectious event**	1.748	1.176–2.598	0.006
**Lower UTI at inf1**	1.923	1.232–3.001	0.004
**Lower respiratory tract infection at inf1**	3.631	1.904–6.924	0.001
**COPD**	2.467	1.376–4.424	0.002

HR—hazard ratio; CI—confidence interval; M0—first visit in the clinic; inf1—first infection; inf2—second infection; inf3—third infection; COPD—chronic obstructive pulmonary disease; UTI—urinary tract infection.

**Table 5:** Independent predictors. Comparison between univariate and multivariate Cox analysis results.

Variable	HR	CI	*p*-Value	HR	CI	*p*-Value
**Age**	1.021	1.005–1.036	0.009	1.034	1.010–1.058	0.004
**Serum ** **creatinine (M0)**	1.262	1.141–1.396	<0.001	1.421	1.203–1.678	<0.001
**Proteinuria (M0)**	1.122	1.028–1.224	0.01	1.241	1.126–1.369	<0.001
**Presence of an infectious event**	1.748	1.176–2.598	0.006	1.705	1.013–2.868	0.04

HR—hazard ratio; CI—confidence interval; M0—first visit in the clinic.

## Data Availability

Due to privacy reasons, the data used in the stiudy is not available.
